# Sustained improvements in patient-reported outcomes after long-term sutimlimab in patients with cold agglutinin disease: results from the CADENZA study open-label extension

**DOI:** 10.1016/j.eclinm.2024.102732

**Published:** 2024-07-18

**Authors:** Alexander Röth, Catherine M. Broome, Wilma Barcellini, Bernd Jilma, Quentin A. Hill, David Cella, Tor Henrik Anderson Tvedt, Masaki Yamaguchi, Irina Murakhovskaya, Michelle Lee, Frank Shafer, Marek Wardęcki, Deepthi Jayawardene, Ronnie Yoo, Jerome Msihid, Ilene C. Weitz

**Affiliations:** a Department of Hematology and Stem Cell Transplantation, West German Cancer Center, University Hospital Essen, University of Duisburg-Essen, Essen, Germany; b Division of Hematology, MedStar Georgetown University Hospital, Washington, DC, USA; c Fondazione IRCCS Ca’ Granda Ospedale Maggiore Policlinico, Milan, Italy; d Department of Clinical Pharmacology, Medical University of Vienna, Vienna, Austria; e Leeds Teaching Hospitals NHS Trust, Leeds, UK; f Department of Medical Social Sciences, Center for Patient-Centered Outcomes, Institute for Public Health and Medicine, Feinberg School of Medicine, Northwestern University, Chicago, IL, USA; g Section for Hematology, Department of Medicine, Haukeland University Hospital, Bergen, Norway; h Ishikawa Prefectural Central Hospital, Japan; i Department of Hematology and Oncology, Albert Einstein College of Medicine/Montefiore Medical Center, Bronx, NY, USA; j Sanofi, Bridgewater, NJ, USA; k Sanofi, Warsaw, Poland; l Sanofi, Cambridge, MA, USA; m Sanofi, Gentilly, France; n Keck School of Medicine of USC, Los Angeles, CA, USA

**Keywords:** Cold agglutinin disease, Sutimlimab, Patient-reported outcomes, Fatigue, Quality of life

## Abstract

**Background:**

Cold agglutinin disease (CAD) is a rare subtype of autoimmune haemolytic anaemia characterised by classical complement pathway-mediated haemolysis, fatigue, and poor quality of life (QoL). Sutimlimab, a C1s inhibitor, rapidly halted haemolysis, and improved patient-reported outcomes (PROs) in patients with CAD in two phase 3 trials (CARDINAL and CADENZA). Here we report PROs from the CADENZA open-label extension (Part B).

**Methods:**

The first patient was enrolled in CADENZA (NCT03347422) in March 2018 (Part A) and the last patient completed the study in December 2021 (Part B). All patients who completed the 26-week Part A were eligible to receive biweekly doses of sutimlimab in Part B for up to 1 year after the last patient completed Part A. PROs were assessed throughout Part B, until the last on-treatment visit with available assessment (LV), and after a 9-week washout.

**Findings:**

In total, 32/39 patients completed Part B; median Part B treatment duration: 99 weeks. Patients switching from placebo to sutimlimab in Part B experienced rapid improvement in Functional Assessment of Chronic Illness Therapy (FACIT)-Fatigue score and other PROs. Sustained, clinically important improvements in FACIT-Fatigue were observed throughout Part B in patients who switched to sutimlimab and those continuing sutimlimab treatment (combined-group mean [SE] change from baseline at LV: 8.8 [2.1]). Similarly, the combined-group mean [SE] change for 12-Item Short Form Health Survey physical (4.9 [1.7]) and mental (4.0 [1.8]) component scores exceeded clinically important changes from baseline at LV. EuroQol visual analogue scale showed consistent and sustained increases from baseline with sutimlimab treatment. Following a 9-week washout, all PROs approached baseline values.

**Interpretation:**

Continued inhibition of the classical complement pathway with sutimlimab results in meaningful long-term improvements in PROs (fatigue and QoL) in patients with CAD.

**Funding:**

10.13039/100004339Sanofi.

**Research in context**

**Evidence before this study**

A PubMed title/abstract search of the terms “Cold agglutinin disease” and “patient-reported outcomes” or “quality of life” limited to English language articles during the last 10 years yields a total of ten publications, including five review articles. Cold agglutinin disease (CAD) impacts the daily lives of many patients, with 90% experiencing fatigue. Patients with CAD are also more likely to have medically attended anxiety or depression. Given the burden of disease, evaluation of patient-reported outcomes (PROs) is an essential part of any new treatment assessment. Sutimlimab is the first therapy indicated for the treatment of CAD. While other medicines are used off-label in CAD, the effect of these on PROs has not been reported. In the phase 3 CARDINAL and CADENZA studies, sutimlimab treatment resulted in improved PROs over 26 weeks (Part A), demonstrating short-term improvement in fatigue, overall health status, and quality of life (QoL).

**Added value of this study**

This is the first assessment of long-term PROs in patients with CAD and no recent history of transfusion. This includes patients who received placebo in CADENZA Part A and switched to sutimlimab during Part B, the open-label extension, and also those who received sutimlimab in Part A and continued with sutimlimab treatment in Part B. The median duration of sutimlimab treatment in Part B was 99 weeks. The study also assesses multiple validated PROs to ensure a clear picture of patient symptoms, overall health status, and QoL. Sustained improvements in all PROs were observed long term with sutimlimab.

**Implications of all the available evidence**

There is increasing interest in PROs, with regulatory bodies recommending their collection and analysis during clinical development. This study supports the burden of disease for patients with CAD, using PROs, and demonstrates a clear benefit of long-term sutimlimab treatment on these outcomes. It also sets a precedent for inclusion of multiple PROs in future trials of new therapies for CAD.

## Introduction

Cold agglutinin disease (CAD) is a rare subtype of autoimmune haemolytic anaemia characterised by chronic haemolysis that is mediated by activation of the classical complement pathway.^1^ In patients with CAD, cold agglutinin autoantibodies preferentially bind to the “I” antigen on red blood cells (RBCs).^1^ These autoantibodies are usually of the immunoglobulin M (IgM) class and can agglutinate RBC at cooler temperatures (≤37 °C) leading to circulatory symptoms such as acrocyanosis or Raynaud's phenomenon.^1^ Furthermore, the IgM-antigen complex binds complement protein 1q (C1q) on the cell surface and initiates activation of the classical complement pathway.^1^^,^^2^ Complement activation leads to extravascular haemolysis, largely through phagocytosis of RBC in the liver. This is the main mechanism of haemolysis in CAD, but intravascular haemolysis via direct complement cascade activation can occur in severe cases, or during episodes of acute inflammation, due to an increased generation of complement and complement activation in the acute phase response.^3^^,^^4^

In addition to increased risk of thromboembolism and decreased life expectancy, CAD impacts patients’ psychological and physical wellbeing and reduces their quality of life (QoL).^5^, ^6^, ^7^, ^8^ This may contribute to additional manifestations of the disease such as depression or anxiety, which can further negatively affect overall mental health and QoL.^9^^,^^10^ Fatigue is the most common symptom reported by patients during an exacerbation of CAD.^10^ Indeed, most patients with CAD report moderate or severe fatigue, tiredness, lack of stamina, and weakness, which occurs daily or multiple times a week, greatly impacting their daily life.^10^

Sutimlimab is a humanised anti-C1s monoclonal antibody designed to target C1s. By selectively inhibiting the classical complement pathway at C1s, sutimlimab retains the immune surveillance functions of the lectin and alternative complement pathways.^11^ Sutimlimab was approved in 2022 by the U.S. Food and Drug Administration, the European Commission, and the Japanese Pharmaceuticals and Medical Devices Agency for use in CAD, and is the only therapy indicated for the treatment of CAD. Two pivotal phase 3 trials have been conducted to investigate the efficacy and safety of sutimlimab in CAD, CARDINAL (a single-arm study in patients with CAD with a recent history of transfusion) and CADENZA (a placebo-controlled study in patients with CAD without a recent history of transfusion). Both studies had a 26-week treatment period (Part A) followed by a long-term extension (Part B), reporting data up to 2 years after the last patient finished Part A of CARDINAL and up to 1 year after the last patient finished Part A of CADENZA.^5^^,^^11^ In both studies, treatment with sutimlimab rapidly inhibited activity of the classical complement pathway, halted haemolysis, increased haemoglobin (Hb) levels, and reduced fatigue in patients with CAD, regardless of recent transfusion history. These beneficial effects were maintained throughout the long-term extension periods.^12^, ^13^, ^14^ In both studies, sutimlimab was generally well tolerated. Overall, the type and frequency of adverse events were generally consistent with the underlying disease indication, reported medical history, and an older medically complex patient population.^11^^,^^12^

Patient-reported outcome (PRO) measures were assessed in both studies, including the Functional Assessment of Chronic Illness Therapy (FACIT)-Fatigue Scale, 12-Item Short Form Health Survey (SF-12), EuroQol 5-Dimension 5-Level questionnaire (EQ-5D-5L), Patient Global Impression of Change (PGIC) and Patient Global Impression of (Fatigue) Severity (PGIS). In CARDINAL, sutimlimab rapidly improved all the examined PROs during the 26-week Part A,^15^ and improvements were maintained up to 2 years after the last patient finished Part A.^16^ Similarly, in CADENZA Part A, treatment with sutimlimab resulted in rapid improvements in all the PRO scores versus placebo-treated patients, with the changes in FACIT-Fatigue and SF-12 representing clinically important changes (CICs) in QoL.^17^

Here we report the long-term effect of sutimlimab treatment on PRO and QoL from Part B, the open-label extension of CADENZA, which aimed to assess the long-term efficacy and safety of sutimlimab in patients with CAD who did not have a recent history of blood transfusion. We also report the effect of discontinuation of sutimlimab treatment on PRO measures, based on data from a 9-week washout period.

## Methods

### Study design and patient population

Full details of the phase 3 CADENZA study have been published elsewhere (Part A,^11^; Part B,^13^). Briefly, CADENZA (NCT03347422) consisted of a 26-week, randomised, placebo-controlled, double-blind treatment period (Part A), conducted from March 2018 to September 2020, with an open-label extension period (Part B), in which patients were receiving sutimlimab for 1 year after the last patient completed Part A, and a 9-week washout period. Data were collected from 53 study locations, including sites in the United States, Australia, Austria, Belgium, Canada, France, Germany, Israel, Italy, Japan, Netherlands, Norway, Spain, and the United Kingdom. The PRO results for Part B are reported here.

Adult patients with a confirmed diagnosis of CAD, Hb ≤10.0 g/dL, bilirubin level above the normal reference range, ferritin level above the lower limit of normal, and no recent history of blood transfusion were eligible. Patients received 26 weeks of either sutimlimab (6.5 g if they weighed <75 kg or 7.5 g if they had a body weight ≥75 kg) or placebo. Study medication was administered by intravenous infusion on day 0, day 7, and every 14 days thereafter through week 25.

Following completion of dosing in the initial 6-month Part A study, patients who continued in Part B received open-label sutimlimab. All patients were dosed with open-label sutimlimab at on-site visits every 2 weeks beginning from week 27. The open-label Part B study was completed 12 months after the last patient completed Part A. All patients who completed Part B proceeded to an end-of-study safety follow-up (SFU) visit approximately 9 weeks after the last administration of study drug. In addition to an SFU, this “washout” visit provided the opportunity to assess the effect of discontinuation of sutimlimab treatment on clinical endpoints. Patients with early termination (ET) were also invited for a follow-up visit 9 weeks after their last dose of sutimlimab. Data from these patients were included in the 9-week “washout” results and combined data are reported for the ET/SFU visit.

The study protocol, any amendments, and patient-informed consent were written according to the Declaration of Helsinki and conducted in accordance with the International Council for Harmonisation Guideline for Good Clinical Practice, and the study was approved by local independent ethics committees or review boards. All patients provided written informed consent for their participation in the study.

### Study PRO endpoints

The primary and secondary efficacy endpoints are covered in the primary reports from Parts A and B of this study.^11^^,^^13^

In CADENZA, a key secondary outcome measure was the mean change from study baseline to treatment assessment timepoint in FACIT-Fatigue score assessed at all scheduled visits. FACIT-Fatigue is a 13-item QoL assessment tool in which patients evaluate their fatigue over the past 7 days by responding to 13 items on a 5-point scale from 0 to 4.^18^ The total score (ranging from 0 to 52, the higher the score, the less the fatigue) is obtained by adding the scores for all questions. The CIC for the FACIT-Fatigue scale is 5 points; this is the lowest score change that indicates a meaningful treatment benefit for patients.^19^^,^^20^

Other secondary outcomes or exploratory QoL endpoints included SF-12 score, EuroQoL visual analogue scale (EQ VAS), the PGIS, the PGIC, and the incidence of solicited anaemia symptoms.

The SF-12 is a 12-item questionnaire measuring eight health domains over the previous 4 weeks. The physical and mental component scores (PCS and MCS) were derived through distinct weighting of these domains, which include general health, physical functioning, effect of physical health on usual role, body pain, overall mental health, vitality, social functioning, and the impact of emotional factors on the patient’s usual role.^21^^,^^22^ Total PCS and MCS were calculated as different weighted means of the eight domains. PCS and MCS are scored on a T-score metric, which has a mean of 50 and standard deviation of 10, referenced to the US general population. A higher score represents improved QoL. The CIC for the PCS and MCS is 3.9 and 2.8, respectively.^23^

The EQ-5D-5L comprises five dimensions (mobility, self-care, usual activities, pain/discomfort and anxiety/depression), and one EQ VAS question. Each dimension has five levels: no problems, slight problems, moderate problems, severe problems, and extreme problems. For the EQ VAS, patients rate their perceived overall health during the current day on a vertical 100-point visual analogue scale, with 0 representing “The worst health you can imagine” and 100 representing “The best health you can imagine”.^24^^,^^25^

The PGIS is a single-item questionnaire with which patients report the severity of their fatigue over the past week (0, None; 1, Mild; 2, Moderate; 3, Severe; 4, Very Severe).^26^

The PGIC is a single-item questionnaire whereby patients report their overall status since the start of the study/baseline (1, Very much improved; 2, Much improved; 3, Minimally improved; 4, No change; 5, Minimally worse; 6, Much worse; 7, Very much worse).^26^

The incidence of solicited symptomatic anaemia (≥1 symptom from presence of fatigue, weakness, shortness of breath, palpitations, light-headedness, and/or chest pain) and change in symptoms by visit (improvement, unchanged, or worsened) was assessed every 2 weeks (beginning at week 27) during Part B prior to administration of study drug. An improvement in total anaemia symptoms is defined as at least one grade reduction in one symptom without any worsening in other remaining symptoms. Conversely, a worsening in total anaemia symptoms is defined as at least one grade increase in one symptom without any improvement in other remaining symptoms.

In Part B of CADENZA, the PRO/QoL instruments were administered at weeks 39, 51, 63, 75, 87, and subsequently at 12-week intervals up to, and including, the last on-treatment visit with available assessment (LV), ie, the last visit while the participant is still taking the study medication, as well as at the ET/SFU visit after the 9-week washout period. Data up to week 87 are shown as they represented the closest visit to the 1-year timepoint (26 weeks [Part A] + 52 weeks [1-year Part B]) where efficacy data were reported.

### Statistical analyses

Details of the sample size considerations for Part A of the study have been published previously.^11^ No power and sample size analysis was conducted for Part B.

The full analysis set (FAS) consisted of all subjects enrolled in Part B who received at least one dose (including partial dose) of study drug. For FACIT-Fatigue, SF-12 and EQ VAS, data were summarised according to treatment received in Part A as well as for the combined Part B population. For the solicited symptoms of anaemia, PGIC and PGIS, data were summarised for the combined group.

Change from baseline of continuous endpoints was summarised by visit using descriptive statistics for the FAS, using the Part A baseline evaluations.

Categorical endpoints were summarised descriptively by counts and percentages. The denominator for all percentages was the number of patients in the FAS who entered Part B.

Analyses were pre-specified, except that data for LVs were post-hoc analyses. No imputation of missing value was performed. All analyses were descriptive. Statistical analysis was performed using SAS® version 9.4 or higher (SAS Institute Inc., Cary, NC).

### Role of the funding source

This study was funded by Sanofi. The study sponsor was involved in study design, the collection, analysis, and interpretation of data, and the writing of the report. All authors had access to primary clinical trial data, and had full editorial control of the manuscript.

## Results

### Baseline demographics

Of the 42 patients enrolled in Part A, 39 patients completed Part A and entered Part B (this included 20 patients previously treated with placebo in Part A and 19 patients who received sutimlimab treatment in Part A). Of the 39 patients enrolled in Part B, 32 (82.1%) patients completed Part B (including 16 patients who received placebo in Part A and switched to sutimlimab in Part B [placebo-to-sutimlimab group] and 16 patients who had received sutimlimab in Part A and continued sutimlimab treatment in Part B [sutimlimab-to-sutimlimab group]). In total, 31 patients completed the SFU visit without ET, and six patients with ET completed the 9-week follow-up visit.

The reasons for discontinuation in Part B for seven (17.9%) patients were treatment-emergent serious adverse events (n = 1), lack of efficacy (n = 3), withdrawal of consent (n = 2), and withdrawal by sponsor (n = 1). The sponsor’s decision to withdraw one patient from the study was due to the patient being unable to attend the end-of-treatment visit within the visit window. Therefore, the last dose of sutimlimab was not administered and the patient was considered to have prematurely discontinued treatment.

One patient out of 32, who completed the full study treatment, had their SFU visit prematurely, and therefore their data were not included in the final analysis because they did not meet the 9-week criteria. One patient out of seven with ET did not complete the 9-week SFU visit due to a fatal TESAE of squamous cell carcinoma of the lung, assessed by the investigator as not related to sutimlimab.

The combined-group median (range) duration of sutimlimab treatment in Part B was 99 weeks (range 22–177); 93 weeks (22–139) for the placebo-to-sutimlimab group, and 118 weeks (43–177) for the sutimlimab-to-sutimlimab group. Patients’ demographics and Part A baseline PRO values for patients entering Part B are shown in Table 1. The mean age in the combined group was 67 years, and most patients were female (79.5%).

**Table 1:** Demographics and baseline PRO values for patients who entered Part B (Full Analysis Set).

Baseline^a^	Treatment in Part A		All patients
	Sutimlimab (n = 19)	Placebo (n = 20)	(N = 39)
Mean (range) age, years	66 (46–88)	68 (51–83)	67 (46–88)
Age ≥ 65 years, n (%)	9 (47)	14 (70)	23 (59)
Female, n (%)	15 (78.9)	16 (80.0)	31 (79.5)
Mean BMI (range), kg/m^2^	24.8 (19–30)	24.0 (18–32)	24.4 (18–32)
Geographic location, n (%)^b^	19	20	39
Europe	12 (63.2)	13 (65.0)	25 (64.1)
North America	3 (15.8)	3 (15.0)	6 (15.4)
Asia	3 (15.8)	2 (10.0)	5 (12.8)
Other	1 (5.3)	2 (10.0)	3 (7.7)
Mean (range) duration of CAD, years	5.8 (0.2–19.0)	6.3 (0.3–20.7)	6.1 (0.2–20.7)
Patients with prior CAD therapy in the past 5 years, n (%)	11 (57.9)	10 (50.0)	21 (53.9)
Mean (SD) FACIT-Fatigue	33.0 (11.7)	33.0 (10.9)	33.0 (11.2)
Mean (SD) SF-12 PCS	43.6 (6.3)	39.0 (7.7)	41.2 (7.4)
Mean (SD) SF-12 MCS	44.8 (9.3)	49.8 (10.0)	47.4 (9.9)
Mean (SD) EQ VAS	62.9 (18.5)	66.0 (18.8)	64.5 (18.4)
Solicited symptoms of anaemia, n (%)			
At least one symptom	15 (78.9)	16 (80.0)	31 (79.5)
Fatigue	15 (78.9)	15 (75.0)	30 (76.9)
Weakness	11 (57.9)	10 (50.0)	21 (53.8)
Shortness of breath	8 (42.1)	12 (60.0)	20 (51.3)
Palpitations	5 (26.3)	4 (20.0)	9 (23.1)
Light-headedness	2 (10.5)	2 (10.0)	4 (10.3)
Chest pain	3 (15.8)	1 (5.0)	4 (10.3)
PGIS Status, n (%)	n = 15	n = 15	n = 30
None	3 (20.0)	2 (13.3)	5 (16.7)
Mild	5 (33.3)	4 (26.7)	9 (30.0)
Moderate	6 (40.0)	7 (46.7)	13 (43.3)
Severe	1 (6.7)	2 (13.3)	3 (10.0)
Very severe	0	0	0

FACIT-Fatigue total score ranges from 0 to 52, the higher score the less fatigue. SF-12 PCS and MCS are scored on a T-score metric, which has a mean of 50 and standard deviation of 10, referenced to the US general population. A higher score represents improved QoL.

BMI, body mass index; EQ VAS, EuroQol visual analogue scale; FACIT, Functional Assessment of Chronic Illness Therapy; MCS, mental component score; PCS, physical component score; PGIS, Patient Global Impression of (Fatigue) Severity; PRO, patient-reported outcome; SD, standard deviation; SF-12, Short form 12-item survey.

a Part A baseline values, for patients entering Part B.

b Europe includes France, Germany, Italy, Norway, and the United Kingdom. North America includes the United States. Asia includes Japan. Other includes Australia and Israel.

### FACIT-Fatigue scores

Upon the switch to open-label treatment in Part B, the mean (SE) FACIT-Fatigue score increased in the placebo-to-sutimlimab group from 33.4 (2.8) at week 26 to 41.1 (2.2) at week 39, the first assessment in Part B, a level comparable to that in patients who received sutimlimab in Part A. In patients treated with placebo, the mean (standard error [SE]) change from baseline was 0.9 (2.8) at week 26. At the first assessment after switch to sutimlimab (week 39) the placebo-to-sutimlimab group mean change from baseline was 8.0 (2.8), exceeding what would be considered a meaningful change in FACIT-Fatigue score (CIC is 5).^19^

Improvements in mean FACIT-Fatigue scores observed in Part A for patients receiving sutimlimab were sustained in Part B for those continuing treatment. The mean (SE) score was 42.2 (2.2) at week 26 and 44.0 (2.1) at week 39. The sutimlimab-to-sutimlimab group mean (SE) change from baseline was 9.3 (2.6) at week 26, and 11.1 (2.9) at week 39, exceeding the CIC of 5 points at both timepoints.^19^

**Fig. 1:**
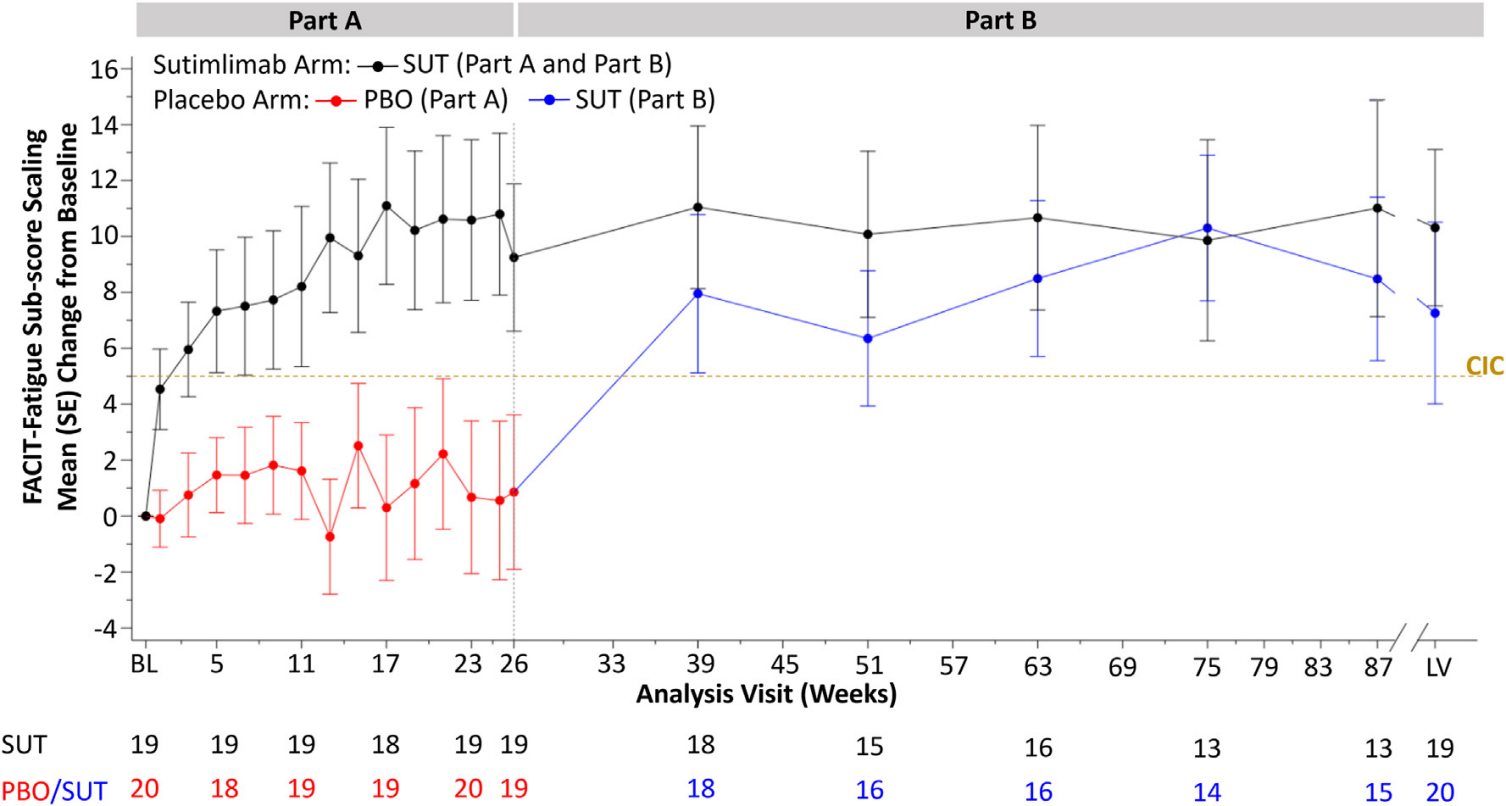
Mean change from baseline in FACIT-Fatigue score (Full Analysis Set). The clinically important change (CIC) reflects the smallest score change that indicates a meaningful treatment benefit for individual patients. Higher scores represent less fatigue. BL, baseline; FACIT, Functional Assessment of Chronic Illness Therapy; LV, last on-treatment visit with available assessment; PBO, placebo; SE, standard error; SUT, sutimlimab.

The mean change from baseline in the FACIT-Fatigue score over time is shown in Fig. 1 for both groups. At the last on-treatment visit (LV), the mean (SE) score was 40.3 (2.8) for the placebo-to-sutimlimab group, and 43.3 (2.1) for the sutimlimab-to-sutimlimab group. The mean (SE) change from baseline was 7.3 (3.3) and 10.3 (2.8), respectively. The mean (SE) change for the combined group (N = 39) was 8.8 (2.1) points.

### SF-12 scores—PCS

**Fig. 2:**
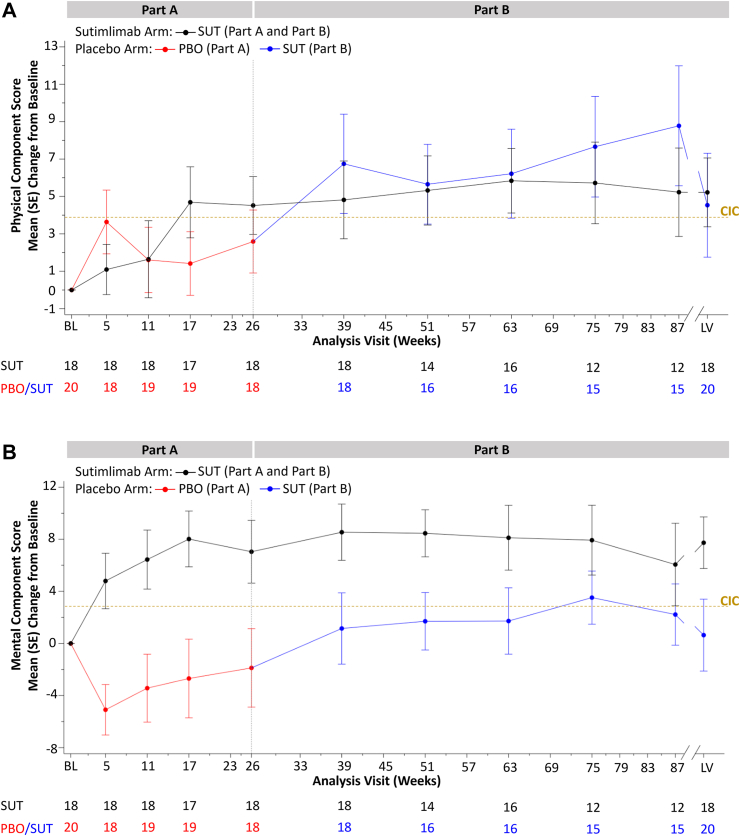
Mean change from baseline in (a) SF-12 PCS and (b) SF-12 MCS (Full Analysis Set). BL, baseline; CIC, clinically important change; LV, last on-treatment visit with available assessment; MCS, mental component score; PCS, physical component score; PBO, placebo; SE, standard error; SF-12, short form 12; SUT, sutimlimab. Higher scores in the SF-12 PCS and MCS represent improved QoL.

The placebo-to-sutimlimab group experienced rapid improvements in SF-12 PCS after switching to sutimlimab (Fig. 2A), with a mean (SE) change from baseline of 6.8 (2.5) at week 39. Improvements were sustained until the end of the study period (mean [SE] change at LV 4.5 [2.8]), although the error bars suggested that some patients' scores did not reach the threshold for a clinically meaningful improvement in SF-12 PCS at several time points.

The sutimlimab-to-sutimlimab group maintained their improved scores from Part A at comparable levels throughout the treatment period in Part B (Fig. 2A). The mean (SE) change from baseline was 4.8 (2.1) at week 39 and 5.2 (1.8) at LV, however, similar to the placebo-to-sutimlimab group, at several time points, the error bars indicated that some patients did not reach the threshold for a clinically meaningful improvement in PCS score.

At the LV, mean (SE) change in PCS from baseline for the combined group (N = 38) was 4.9 (1.7) points, exceeding the CIC of 3.9.

### SF-12 scores—MCS

The sutimlimab-to-sutimlimab group showed an improvement in SF-12 MCS that exceeded the CIC of 2.8 at all post-baseline visits in Part A, specifically weeks 5, 11, 17, and 26 (Fig. 2B). Throughout the Part B treatment period, the mean change from baseline also exceeded the CIC in this group, with a mean (SE) change of 7.7 (2.0) at LV.

Conversely, the mean (SE) MCS score for patients on placebo worsened by 5.1 (1.8) at the first visit in Part A of the study and remained below baseline until the end of Part A (Fig. 2B). Upon switching to sutimlimab in Part B, the mean for the placebo-to-sutimlimab group improved, exceeding the baseline mean at week 39 (the first assessment after switching) for the first time in the study (mean [SE] change from baseline 1.2 [2.6]). The mean change in MCS eventually exceeded the CIC of 2.8 in this group at week 75 (mean [SE] change from baseline 3.5 [1.8]). However, this was not sustained until LV, (mean [SE] change from baseline 0.6 [2.8]), and the error bars indicated that some patients did not achieve clinically meaningful improvements in MCS in the placebo-to-sutimlimab group.

At the LV, the mean (SE) change in MCS for the combined group (N = 38) was 4.0 (1.8) points, which exceeded the CIC of 2.8 points. Of the individual subscale domains of the SF-12, most notable and sustained improvements with sutimlimab treatment in the combined group were observed on physical health, physical functioning, role physical, and vitality (Supplementary Table S1).

### EQ VAS score

**Fig. 3:**
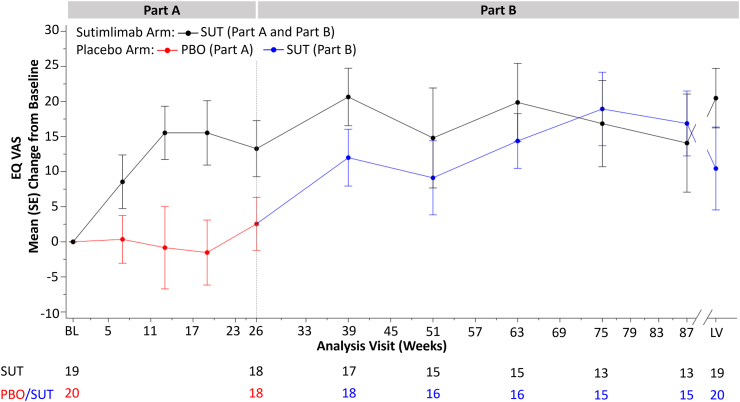
Mean change from baseline in EQ VAS score (Full Analysis Set). BL, baseline; EQ VAS, EuroQol Visual Analogue Scale; LV, last on-treatment visit with available assessment; PBO, placebo; SE, standard error; SUT, sutimlimab. Higher scores in the EQ VAS represent better health/QoL.

The placebo-to-sutimlimab group demonstrated rapid improvement in EQ VAS when switched to sutimlimab (Fig. 3), with a mean (SE) change from baseline of 12.0 (3.8) at week 39. This was sustained throughout the duration of the study, with a mean (SE) change from baseline of 10.5 (5.9) at LV.

Similarly, the sutimlimab-to-sutimlimab group maintained improved EQ VAS scores through Part B, with a mean (SE) change from baseline of 20.7 (3.9) at week 39 and 20.5 (4.2) at LV (Fig. 3). At LV the mean (SE) change in EQ VAS for the combined group (N = 39) was 15.3 (3.7) points.

Of the different dimensions of the EQ-5D-5L, most improvements with sutimlimab treatment in the combined group were observed on mobility, doing usual activities, and anxiety/depression (Supplementary Table S2).

### PGIS and PGIC

**Fig. 4:**
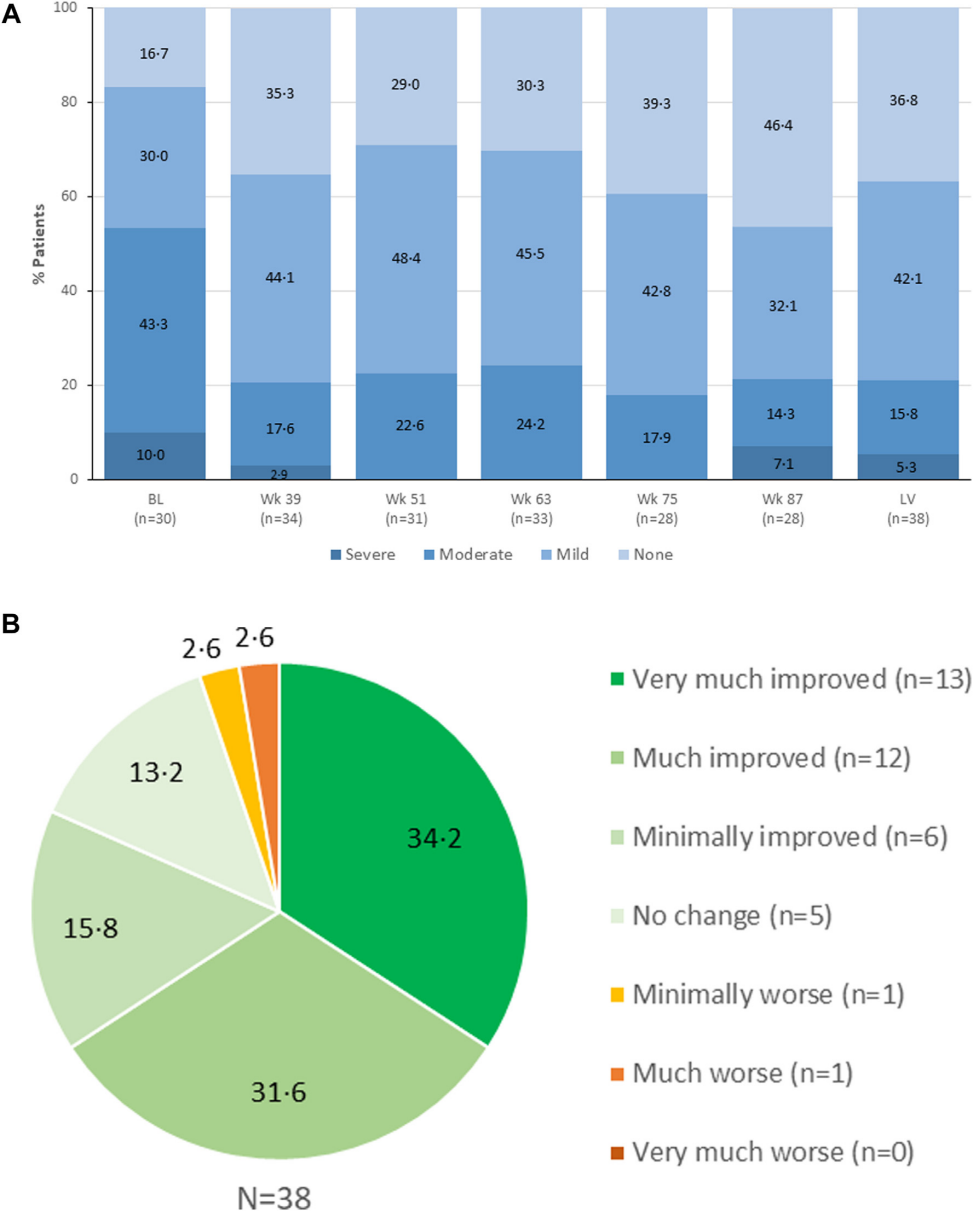
Percent patients per response category for (a) Patient Global Impression of (Fatigue) Severity (PGIS) over time and (b) Patient Global Impression of Change (PGIC) overall at the last on-treatment visit (LV) with available assessment (Full Analysis Set). BL, baseline; LV, last on-treatment visit with available assessment.

It was previously reported that at the end of Part A, patients in the placebo group felt significantly more fatigued than patients who had been treated with sutimlimab for 26 weeks.^17^ Upon switching to sutimlimab, patients in the placebo-to-sutimlimab group saw an improvement in PGIS, with 70.6% reporting “None” or “Mild” fatigue at week 39, almost reaching the levels reported in the sutimlimab-to-sutimlimab group (88.3%). In the combined group, 79.4% of patients receiving sutimlimab at week 39 indicated “None” or “Mild” fatigue, an improvement from 46.7% at baseline, which stayed consistent throughout Part B (78.9% at LV) (Fig. 4A). The improvement in fatigue severity was sustained over time, with proportions among each categorical response varying only slightly at different timepoints (Supplementary Table S3).

At the end of Part A (week 26), 73.7% of patients in the sutimlimab group reported an improvement in their overall status in the PGIC since the start of the study, compared with 31.6% in the placebo group.^17^ By the first assessment timepoint in Part B (week 39), a large majority of patients in both groups reported improvement (93.8% in the placebo-to-sutimlimab group and 82.4% in the sutimlimab-to-sutimlimab group). PGIC, reporting impression of overall change since the start of the study, indicated 81.6% of the combined group perceived at least minimal improvement at LV; among those, 34.2% reported “Very much improved” (Fig. 4B). Improvement (minimal, much, or very much) relative to baseline was observed in >70% of patients at all timepoints (Supplementary Table S3).

### Solicited symptoms of anaemia

The incidence of solicited symptomatic anaemia (fatigue, weakness, shortness of breath, palpitations/fast heartbeat, light-headedness, and/or chest pain) was assessed at baseline and followed up regularly throughout the study. At Part A baseline, 31 (79.5%) patients reported ≥1 solicited symptom of anaemia of any grade. In the overall Part B population, the percentage of patients reporting individual solicited symptoms of anaemia decreased during the study with sutimlimab treatment, with 35.7% patients in the combined group at week 87 reporting ≥1 symptom (Supplementary Fig. S1a). Improvement in at least one solicited symptom of anaemia versus baseline was observed in 22 (78.6%) patients at week 87 (improvement defined as at least one grade reduction in one symptom without any worsening in other remaining symptoms). The most frequently reported symptoms of anaemia were fatigue, weakness, and shortness of breath. More patients reported improvements in fatigue, weakness, and shortness of breath than other symptoms (Supplementary Fig. S1b).

### Effect of discontinuation of sutimlimab treatment

At ET/SFU, 9 weeks after the last dose of sutimlimab, in the analysis of the combined group, mean Hb decreased from the last available value during treatment to a level comparable to the baseline value. Similarly, haemolysis increased, as evidenced by an increase in mean total bilirubin and other haemolytic markers from the end of treatment, to values comparable to the baseline value.^13^ FACIT-Fatigue, SF-12 PCS and MCS scores, and EQ VAS scores similarly worsened after the 9-week washout in the combined group, decreasing to scores comparable to baseline values (Table 2, Supplementary Fig. S2).

**Table 2:** Combined Group mean values of PRO measures at baseline, and in Part B last on-treatment visit (LV) and early termination/safety follow-up (ET/SFU) 9 weeks post last sutimlimab dose (Full Analysis Set).

Mean PRO score (SE)	Baseline	LV	ET/SFU	Change at ET/SFU relative to baseline
FACIT-Fatigue	n = 39 33.0 (1.8)	n = 39 41.7 (1.7)	n = 37 31.3 (2.3)	n = 37 −1.4 (1.9)
SF-12 PCS	n = 38 41.2 (1.2)	n = 39 45.8 (1.5)	n = 37 39.6 (1.7)	n = 36 −2.0 (1.8)
SF-12 MCS	n = 38 47.4 (1.6)	n = 39 51.8 (1.4)	n = 37 47.0 (2.0)	n = 36 −0.2 (1.9)
EQ VAS	n = 39 64.5 (3.0)	n = 39 79.8 (2.9)	n = 37 63.3 (3.8)	n = 37 −0.2 (2.7)

EQ VAS, EuroQol visual analogue scale; ET/SFU, early termination/safety follow-up 9 weeks post last sutimlimab dose; FACIT, Functional Assessment of Chronic Illness Therapy; LV, last on-treatment visit with available assessment; MCS, mental component score; PCS, physical component score; PRO, patient-reported outcome; SE, standard error; SF-12, Short form 12-item survey.

FACIT-Fatigue total score ranges from 0 to 52, the higher score the less fatigue. SF-12 PCS and MCS are scored on a T-score metric, which has a mean of 50 and standard deviation of 10, referenced to the US general population. A higher score represents improved QoL.

The proportion of patients reporting favourable PGIC status decreased from LV values after washout. At week 39, LV, and ET/SFU, respectively, “Improved” status was reported by 87.8%, 81.6%, and 59.4% of patients; “Unchanged” was reported by 9.1%, 13.2%, and 16.2% of patients; and “Worsened” was reported by 3.0%, 5.2%, and 24.3% of patients (Supplementary Table S1).

Similarly, in the combined group PGIS assessments were less favourable after washout. At week 39, LV, and ET/SFU, respectively, the percentages of patients reporting fatigue severity were as follows: “None” 35.3%, 36.8%, and 16.2%; “Mild” 44.1%, 42.1%, and 21.6%; “Moderate” 17.6%, 15.8%, and 32.4%; “Severe” or “Very severe” 2.9%, 5.3%, and 29.7% (Supplementary Table S1).

## Discussion

Part A of the CADENZA study showed that 26 weeks of treatment with sutimlimab inhibited activity in the classical complement pathway, halted haemolysis, increased Hb levels, and reduced fatigue in patients with CAD, relative to placebo.^11^ In Part B of CADENZA, treatment with sutimlimab continued to inhibit haemolysis and improve anaemia and QoL for up to 1 year after the last patient finished Part A.^13^ Responses were similar in patients administered sutimlimab throughout the study and those who switched to sutimlimab in Part B. Sutimlimab was generally well tolerated throughout the study.^13^ Overall, the type and frequency of treatment-emergent adverse events were generally consistent with those expected for this patient population.

The results described in the current paper demonstrated that improvements seen with sutimlimab during Part A in fatigue and overall QoL^11^^,^^17^ were maintained with continued sutimlimab treatment in Part B, for up to 1 year after the last patient finished Part A. Patients previously treated with placebo in Part A demonstrated rapid improvements in PRO measures when switched to sutimlimab in Part B. Throughout Part B, most of the combined group reported improved QoL compared with baseline.

In the sutimlimab-to-sutimlimab group, the change in FACIT-Fatigue and SF-12 PCS scores exceeded the CIC throughout Part B, and the FACIT-Fatigue score was within the normal range when referenced with the general population.^19^^,^^23^^,^^27^ In the placebo-to-sutimlimab group, the change in FACIT-Fatigue and SF-12 PCS scores exceeded the CIC by week 39 and remained above the CIC until the last visit. The placebo-to-sutimlimab group experienced a worsening in the SF-12 MCS during Part A. Therefore, upon treatment with sutimlimab in Part B, the SF-12 MCS started from a point lower than baseline. Scores improved slowly and exceeded the CIC only at week 75. The SF-12 comprises eight domains, and improvements on those relating to mental health and emotional factors were less noticeable or slower to take effect. It is conceivable that once a patient feels their mental health has been impacted, resolution of their symptoms may not necessarily mirror improvement in their emotional wellbeing. However, it would seem that fatigue does not fit into this category, since rapid and sustained improvements in FACIT-Fatigue were observed with sutimlimab treatment, although not all scores in the sutimlimab-to-placebo group reached the levels of those who had received sutimlimab throughout the study. Additionally, all SF-12 domain scores contribute to the scoring of both the PCS and MCS, albeit in different directions. For example, a positive mental health impact on fatigue would somewhat deflate a positive PCS score, as seen in the initial study period for the sutimlimab group. The SF-12 scoring algorithm is based on the simplified assumption that physical and mental health are uncorrelated, and this should be considered when interpreting SF-12 data.^28^

The majority of patients reported no or mild fatigue at LV, and similarly most patients also considered that their clinical status had improved relative to baseline. Interestingly, 60% of patients still reported “improved” PGIC after 9 weeks off sutimlimab treatment. The PGIC reflects a patient’s overall wellbeing but has an inherent bias since it requires patients to compare current health status with that at baseline. With a median sutimlimab treatment duration in Part B of 99 weeks, baseline status may be difficult to recall for some patients. Other PROs more accurately represent the current health status, requiring patients to recall only the last day, week, or month. For example, the PGIS, which reflects a patient’s fatigue severity during the past week, may be a more accurate measure.

In the 9-week SFU period following the final dose of sutimlimab, the PRO scores worsened and approached pre-treatment values. As such, PROs generally track the clinical data, which indicate that haemolytic anaemia recurred in most patients once treatment was discontinued, with mean levels of haemolytic markers and Hb approaching pre-treatment values upon cessation of sutimlimab.^13^ The majority of the adverse events reported during the washout period were due to exacerbation of underlying CAD.^13^

Strengths of this study are the use of validated PRO instruments (FACIT-Fatigue and the SF-12) as a measure of patient QoL, and that it adds new, valuable information on how treatment with sutimlimab can sustain improvements in PROs in CAD up to 1 year after the last patient finished Part A. Long-term PRO data are limited in patients with CAD, and lacking in the assessment of treatments for CAD, such as rituximab. To our knowledge, sutimlimab is the first treatment for CAD in which PRO and QoL data have been investigated. Longer-term real-world PRO data for sutimlimab are being collected in CADENCE, a multinational, multicentre, observational, prospective, longitudinal registry for patients with CAD.^29^

A study limitation is the small sample size. However, this reflects the fact that CAD is a rare disease and is in keeping with studies of other rare autoimmune diseases in the literature. While most analyses were prospectively defined, a further limitation is that they were descriptive and that the analyses pertaining to LV were post hoc. In addition, since all patients were aware they were receiving sutimlimab in Part B of the study, any improvements observed in patients switching from placebo to sutimlimab should be interpreted with caution. This is because it is possible that patients may have felt better knowing they were receiving the study drug, which could partially explain any improvements in PROs observed in Part B. However, improvements were also observed in more objective measures of disease activity (published in a separate manuscript) including improvements in Hb and bilirubin levels, which are markers of anaemia and haemolysis, respectively. Another potential confounding factor may be that patients were not blinded to their Hb and bilirubin levels, which are objective measures of anaemia and haemolysis, respectively. Although, it should be noted that during study visits, questionnaires were completed before Hb or bilirubin values were communicated to the patients, thus reducing the potential influence of laboratory data on self-reported QoL.

In conclusion, CADENZA Part B has shown that, in addition to improving haematological parameters in patients with CAD and without recent history of transfusion, continued inhibition of the classical complement pathway via treatment with sutimlimab results in meaningful long-term benefits to fatigue and patient-reported QoL. These results support the effectiveness of targeting the classical complement pathway (inhibition of C1s) in the management of CAD.

## Contributors

All authors had access to primary clinical trial data, had full editorial control of the manuscript, and provided their final approval of all content.

## Data sharing statement

Qualified researchers may request access to patient-level data and related study documents, including the clinical study report, study protocol with any amendments, blank case report form, statistical analysis plan, and dataset specifications. Patient-level data will be anonymised and study documents will be redacted to protect the privacy of our trial participants. The study protocol and statistical analysis plan are also available via clinicaltrials.gov: Prot_000.pdf (clinicaltrials.gov) and SAP_001.pdf (clinicaltrials.gov), respectively. Further details on Sanofi’s data sharing criteria, eligible studies, and process for requesting access can be found at: https://www.vivli.org/.

## Declaration of interests

**AR** has received consultancy fees from Alexion Pharmaceuticals, Inc, Apellis Pharmaceuticals, Bioverativ, a Sanofi company, Novartis, Roche, and Sanofi; honoraria from Alexion, Amgen, Apellis, Novartis, Roche, Sanofi and Sobi, and advisory board fees from Alexion, Amgen, Apellis, Bioverativ, Novartis, Roche, Sanofi, and Sobi.

**CMB** has received research support from Alexion, Argenx, Electra, Novartis, and Sanofi; honoraria from Alexion, Argenx, and Sanofi; and advisory board fees from Argenx, Novartis, and Sanofi.

**WB** has received research support from Alexion; honoraria from Agios, Alexion, Apellis, Biocryst, Incyte, Janssen, Momenta, Novartis, Sanofi, and Sobi; and advisory board fees from Alexion, Novartis, Roche, Sanofi, and Sobi.

**BJ** has received reimbursement for travel costs related to scientific advice and scientific presentations from Sanofi.

**QAH** has received research support from Alexion; consultancy fees from Amgen, Argenx, Gliknik, Grifols, Incyte, Immunovant, Janssen, Novartis, ReAlta, Sanofi, and Sobi; and honoraria from Amgen, Argenx, Bioverativ, Gliknik, Grifols, Incyte, Immunovant, Janssen, Novartis, ReAlta, Sanofi, Shire, and Sobi.

**DC** has received research support to his institution from Astellas and consulting fees from Sanofi.

**THAT** has received honoraria from Ablynx, Alexion, and Novartis.

**MY** has no disclosures.

**IM** has received consultancy fees and honoraria from Alexion, Apellis, Momenta/Janssen, Novartis, Rigel, Sanofi.

**ICW** has received consultancy fees from Alexion, Apellis, Novartis, and Biocryst; and honoraria from Alexion.

**ML, FS, MW, DJ, RY** and **JM** are Sanofi employees and may hold stock and/or stock options in the company.
