# Supplementary material for: Sustained improvements in patient-reported outcomes after long-term sutimlimab in patients with cold agglutinin disease: results from the CADENZA study open-label extension
Source: eClinicalMedicine. 2024 Jul 18;74:102732. doi: 10.1016/j.eclinm.2024.102732 (PMC11304688; doi:10.1016/j.eclinm.2024.102732)
Supplement: Supplementary Figs. S1 and S2 and Tables S1–S3 [file mmc1.pdf]

**Sustained improvements in patient-reported outcomes (PROs) after long-term sutimlimab use in patients with cold agglutinin disease: Part B of the phase 3 CADENZA Study**

## Supplementary Appendix

### Table of Contents

*Table S1*: Summary of SF-12 Subscale Scores by Visit in the Combined Part B Population (Full Analysis Set).. 2

*Table S2*: Summary of EQ-5D-5L Domain Responses by Visit in the Combined Part B Population (Full Analysis Set). 3

*Table S3*: Summary of Patient's Global Impression of (Fatigue) Severity (PGIS) and Patient's Global Impression of Change (PGIC) by Visit in the Combined Part B Population (Full Analysis Set). 5

*Figure S1*: Incidence and change in solicited symptoms of anaemia. (a) Reduction in incidence of solicited symptoms of anaemia from Part A baseline (n=39) to week 87 (n=28) with sutimlimab treatment. (b) Improvements in solicited symptoms of anaemia vs baseline at weeks 27 (n=37) and 87 (n=28) with sutimlimab treatment (Full Analysis Set). 6

*Figure S2*: Effect of discontinuation of treatment on (a) mean FACIT-Fatigue scores, (b) mean EQ-VAS scores, and (c, d) mean SF-12 PCS and MCS scores. PRO scores at baseline, last available on-treatment visit (LV), and end-of-treatment/safety follow-up visits (ET/SFU) (Full Analysis Set). 7

**Table S1: Summary of SF-12 Subscale Scores by Visit in the Combined Part B Population (Full Analysis Set).**

| | Part A | -------------------- | -------------------- | -------------------- | Part B | -------------------- | -------------------- | -------------------- |
|---|---|---|---|---|---|---|---|---|
| **Domain**<br>**Mean (SE) score** | **Baseline**<br>**(n=39)** | **Week 39**<br>**(n=36)** | **Week 51**<br>**(n=31)** | **Week 63**<br>**(n=33)** | **Week 75**<br>**(n=28)** | **Week 87**<br>**(n=28)** | **LV**<br>**(n=39)** | **ET/SFU**<br>**(n=37)** |
| **Physical Health** | 41·3 (1·5) | 47·5 (1·7) | 47·8 (1·7) | 48·5 (1·6) | 49·9 (1·6) | 48·1 (1·5) | 47·1 (1·6) | 40·0 (1·9) |
| **Physical Functioning** | 39·1 (1·6) | 46·4 (1·4) | 47·7 (1·4) | 46·3 (1·5) | 47·8 (1·6) | 45·3 (2·1) | 45·0 (1·7) | 38·1 (1·7) |
| **Role Physical** | 40·5 (1·4)* | 47·4 (1·4) | 47·0 (1·5) | 46·8 (1·3) | 49·2 (1·5) | 48·4 (1·9) | 46·1 (1·5) | 39·7 (1·8) |
| **Bodily Pain** | 51·1 (1·3)* | 50·0 (1·4) | 49·3 (1·6) | 50·6 (1·6) | 49·7 (1·7) | 49·0 (1·9) | 50·3 (1·6) | 46·5 (2·0) |
| **Role Emotional** | 44·2 (1·8)* | 47·6 (1·4) | 47·9 (1·8) | 48·6 (1·7) | 50·0 (1·9) | 46·8 (2·2) | 47·0 (1·9) | 42·1 (2·3) |
| **Vitality** | 43·8 (1·7) | 54·8 (1·6) | 53·5 (1·6) | 54·4 (1·7) | 55·4 (1·5) | 54·0 (1·7) | 53·1 (1·5) | 45·3 (2·0) |
| **Mental Health** | 49·8 (1·6) | 53·2 (1·6) | 54·6 (1·3) | 52·2 (1·6) | 56·0 (1·3) | 52·7 (2·0) | 53·3 (1·5) | 48·1 (1·9) |
| **Social Functioning** | 42·8 (1·6) | 49·0 (1·5) | 49·2 (1·6) | 49·1 (1·7) | 50·2 (1·7) | 49·3 (1·8) | 46·9 (1·7) | 42·2 (1·9) |

*For these subscales, n=38 at baseline.
ET/SFU, early termination/safety follow-up 9 weeks post last sutimlimab dose; SE, standard error; SF-12, short-form 12-item health survey.

**Table S2: Summary of EQ-5D-5L Domain Responses by Visit in the Combined Part B Population (Full Analysis Set).**

| | Part A | ---------------------------- | ---------------------------- | ------------------------- | Part B | ------------------------- | ---------------------------- | ---------------------------- |
|---|---|---|---|---|---|---|---|---|
| **Domain/Response** | **Baseline (n=39)** | **Week 39 (n=35)** | **Week 51 (n=31)** | **Week 63 (n=32)** | **Week 75 (n=28)** | **Week 87 (n=28)** | **LV (n=39)** | **ET/SFU (n=37)** |
| **Mobility** (any problems walking about), n (%) | | | | | | | | |
| No problems | 14 (35·9) | 24 (68·6) | 20 (64·5) | 20 (62·5) | 19 (67·9) | 19 (67·9) | 26 (66·7) | 14 (37·8) |
| Slight problems | 16 (41·0) | 8 (22·9) | 7 (22·6) | 8 (25·0) | 6 (21·4) | 4 (14·3) | 7 (17·9) | 11 (29·7) |
| Moderate problems | 9 (23·1) | 2 (5·7) | 3 (9·7) | 3 (9·4) | 2 (7·1) | 3 (10·7) | 4 (10·3) | 7 (18·9) |
| Severe problems | 0 | 1 (2·9) | 1 (3·2) | 0 | 0 | 1 (3·6) | 1 (2·6) | 4 (10·8) |
| Unable to walk | 0 | 0 | 0 | 1 (3·1) | 1 (3·6) | 1 (3·6) | 1 (2·6) | 1 (2·7) |
| **Self-Care** (any problems washing or dressing myself), n (%) | | | | | | | | |
| No problems | 36 (92·3) | 33 (94·3) | 28 (90·3) | 30 (93·8) | 25 (89·3) | 25 (89·3) | 36 (92·3) | 30 (81·1) |
| Slight problems | 3 (7·7) | 0 | 2 (6·5) | 0 | 2 (7·1) | 2 (7·1) | 2 (5·1) | 3 (8·1) |
| Moderate problems | 0 | 0 | 1 (3·2) | 1 (3·1) | 1 (3·6) | 0 | 0 | 2 (5·4) |
| Severe problems | 0 | 1 (2·9) | 0 | 0 | 0 | 0 | 0 | 1 (2·7) |
| Unable to wash or dress | 0 | 1 (2·9) | 0 | 1 (3·1) | 0 | 1 (3·6) | 1 (2·6) | 1 (2·7) |
| **Usual Activities** (any problems doing usual activities), n (%) | | | | | | | | |
| No problems | 18 (46·2) | 22 (62·9) | 20 (64·5) | 19 (59·4) | 18 (64·3) | 16 (57·1) | 24 (61·5) | 12 (32·4) |
| Slight problems | 13 (33·3) | 8 (22·9) | 9 (29·0) | 10 (31·3) | 8 (28·6) | 9 (32·1) | 9 (23·1) | 12 (32·4) |
| Moderate problems | 7 (17·9) | 5 (14·3) | 1 (3·2) | 2 (6·3) | 2 (7·1) | 3 (10·7) | 5 (12·8) | 7 (18·9) |
| Severe problems | 1 (2·6) | 0 | 1 (3·2) | 1 (3·1) | 0 | 0 | 0 | 3 (8·1) |
| Unable to do usual activities | 0 | 0 | 0 | 0 | 0 | 0 | 1 (2·6) | 3 (8·1) |
| **Pain/Discomfort** (any pain or discomfort), n (%) | | | | | | | | |
| None | 19 (48·7) | 18 (51·4) | 14 (45·2) | 17 (53·1) | 14 (50·0) | 11 (39·3) | 23 (59·0) | 12 (32·4) |
| Slight | 11 (28·2) | 12 (34·3) | 12 (38·7) | 9 (28·1) | 11 (39·3) | 12 (42·9) | 11 (28·2) | 13 (35·1) |
| Moderate | 7 (17·9) | 4 (11·4) | 3 (9·7) | 4 (12·5) | 2 (7·1) | 4 (14·3) | 3 (7·7) | 7 (18·9) |
| Severe | 2 (5·1) | 1 (2·9) | 1 (3·2) | 2 (6·3) | 1 (3·6) | 1 (3·6) | 2 (5·1) | 5 (13·5) |
| Extreme | 0 | 0 | 1 (3·2) | 0 | 0 | 0 | 0 | 0 |
| **Anxiety/Depression** (any anxiety or depression, n (%) | | | | | | | | |

| | | | | | | | | |
|---|---|---|---|---|---|---|---|---|
| None | 19 (48·7) | 18 (51·4) | 21 (67·7) | 22 (68·8) | 22 (78·6) | 21 (75·0) | 25 (64·1) | 16 (43·2) |
| Slight | 17 (43·6) | 14 (40·0) | 8 (25·8) | 8 (25·0) | 6 (21·4) | 5 (17·9) | 12 (30·8) | 17 (45·9) |
| Moderate | 3 (7·7) | 3 (8·6) | 2 (6·5) | 2 (6·3) | 0 | 1 (3·6) | 1 (2·6) | 4 (10·8) |
| Severe | 0 | 0 | 0 | 0 | 0 | 1 (3·6) | 1 (2·6) | 0 |
| Extreme | 0 | 0 | 0 | 0 | 0 | 0 | 0 | 0 |

ET/SFU, early termination/safety follow-up 9 weeks post last sutimlimab dose; EQ-5D-5L, EuroQol 5-Dimension 5-Level questionnaire.

Percentages based on number of patients in the Full Analysis Set in Part B who completed the EQ-5D-5L at the specified visit.

**Table S3: Summary of Patient's Global Impression of (Fatigue) Severity (PGIS) and Patient's Global Impression of Change (PGIC) by Visit in the Combined Part B Population (Full Analysis Set).**

| | Part A | --------------------------------------------------------------------------Part B-------------------------------------------------------------------------- | | | | | | |
|---|---|---|---|---|---|---|---|---|
| **PGIS status, n (%)** | **Baseline (n=30)** | **Week 39 (n=34)** | **Week 51 (n=31)** | **Week 63 (n=33)** | **Week 75 (n=28)** | **Week 87 (n=28)** | **LV (n=38)** | **ET/SFU (n=37)** |
| None | 5 (16·7) | 12 (35·3) | 9 (29·0) | 10 (30·3) | 11 (39·3) | 13 (46·4) | 14 (36·8) | 6 (16·2) |
| Mild | 9 (30·0) | 15 (44·1) | 15 (48·4) | 15 (45·4) | 12 (42·9) | 9 (32·1) | 16 (42·1) | 8 (21·6) |
| Moderate | 13 (43·3) | 6 (17·6) | 7 (22·6) | 8 (24·2) | 5 (17·9) | 4 (14·3) | 6 (15·8) | 12 (32·4) |
| Severe | 3 (10·0) | 1 (2·9) | 0 | 0 | 0 | 2 (7·1) | 2 (5·3) | 8 (21·6) |
| Very Severe | 0 | 0 | 0 | 0 | 0 | 0 | 0 | 3 (8·1) |
| **PGIC Status, n (%)** | | **Week 39 (n=33)** | **Week 51 (n=31)** | **Week 63 (n=33)** | **Week 75 (n=28)** | **Week 87 (n=28)** | **LV (n=38)** | **ET/SFU (n=37)** |
| Very much improved | | 7 (21·2) | 8 (25·8) | 8 (24·2) | 12 (42·9) | 14 (50·0) | 13 (34·2) | 6 (16·2) |
| Much improved | | 14 (42·4) | 9 (29·0) | 12 (36·4) | 5 (17·9) | 3 (10·7) | 12 (31·6) | 11 (29·7) |
| Minimally improved | | 8 (24·2) | 6 (19·4) | 5 (15·2) | 6 (21·4) | 3 (10·7) | 6 (15·8) | 5 (13·5) |
| No change | | 3 (9·1) | 7 (22·6) | 7 (21·2) | 5 (17·9) | 5 (17·9) | 5 (13·2) | 6 (16·2) |
| Minimally worse | | 1 (3·0) | 0 | 0 | 0 | 2 (7·1) | 1 (2·6) | 3 (8·1) |
| Much worse | | 0 | 1 (3·2) | 1 (3·0) | 0 | 1 (3·6) | 1 (2·6) | 6 (16·2) |
| Very much worse | | 0 | 0 | 0 | 0 | 0 | 0 | 0 |

ET/SFU, early termination/safety follow-up 9 weeks post last sutimlimab dose; LV, last on-treatment visit with available assessment; PGIC, Patient's Global Impression of Change; PGIS, Patient's Global Impression of (Fatigue) Severity.

**Figure S1: Incidence and change in solicited symptoms of anaemia. (a) Reduction in incidence of solicited symptoms of anaemia from Part A baseline (n=39) to week 87 (n=28) with sutimlimab treatment. (b) Improvements in solicited symptoms of anaemia vs baseline at weeks 27 (n=37) and 87 (n=28) with sutimlimab treatment (Full Analysis Set).**

**a)**

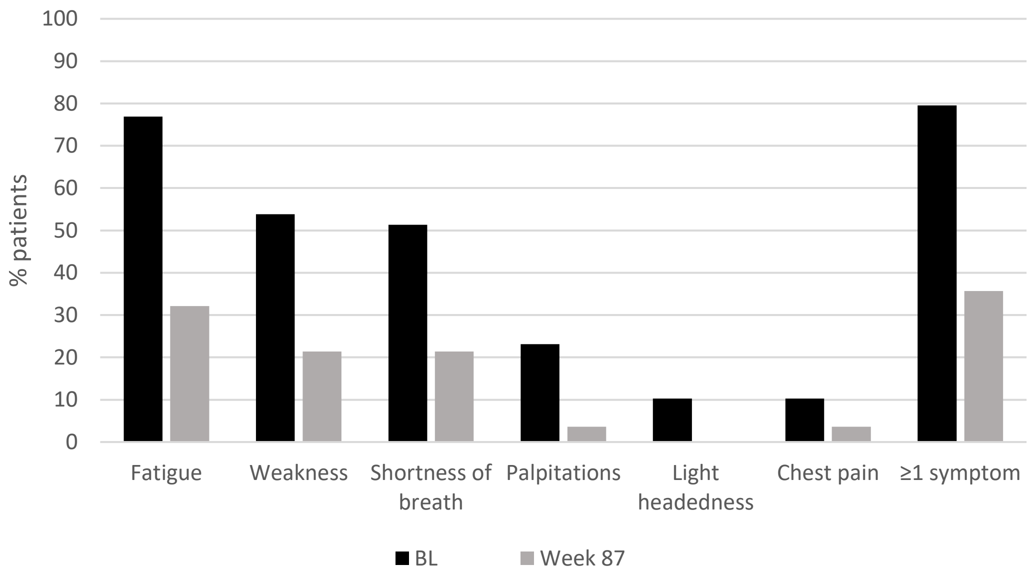


**b)**

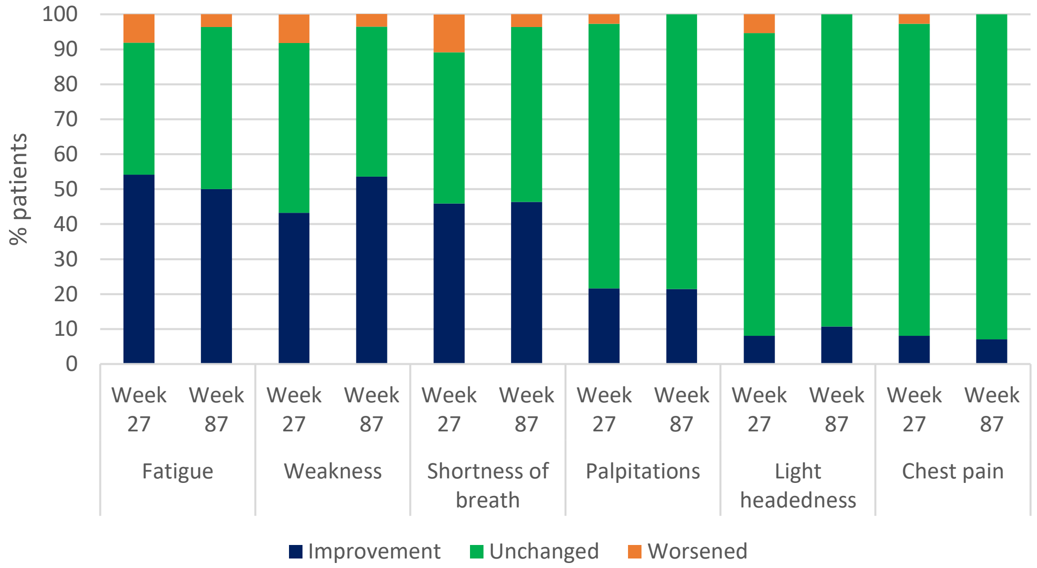


An improvement is defined as at least one grade reduction in one symptom without any worsening in other remaining symptoms. A worsening is defined as at least one grade increase in one symptom without any improvement in other remaining symptoms.
Week 27 represents the last timepoint in Part A before patients entered Part B. Data at week 87 are shown as they were the closest visit to the 1-year timepoint (26 weeks [Part A] + 52 weeks [1- year Part B]) where efficacy data were reported.
BL, baseline.

**Figure S2: Effect of discontinuation of treatment on (a) mean FACIT-Fatigue scores, (b) mean EQ-VAS scores, and (c, d) mean SF-12 PCS and MCS scores. PRO scores at baseline, last available on-treatment visit (LV), and end-of-treatment/safety follow-up visits (ET/SFU) (Full Analysis Set).**

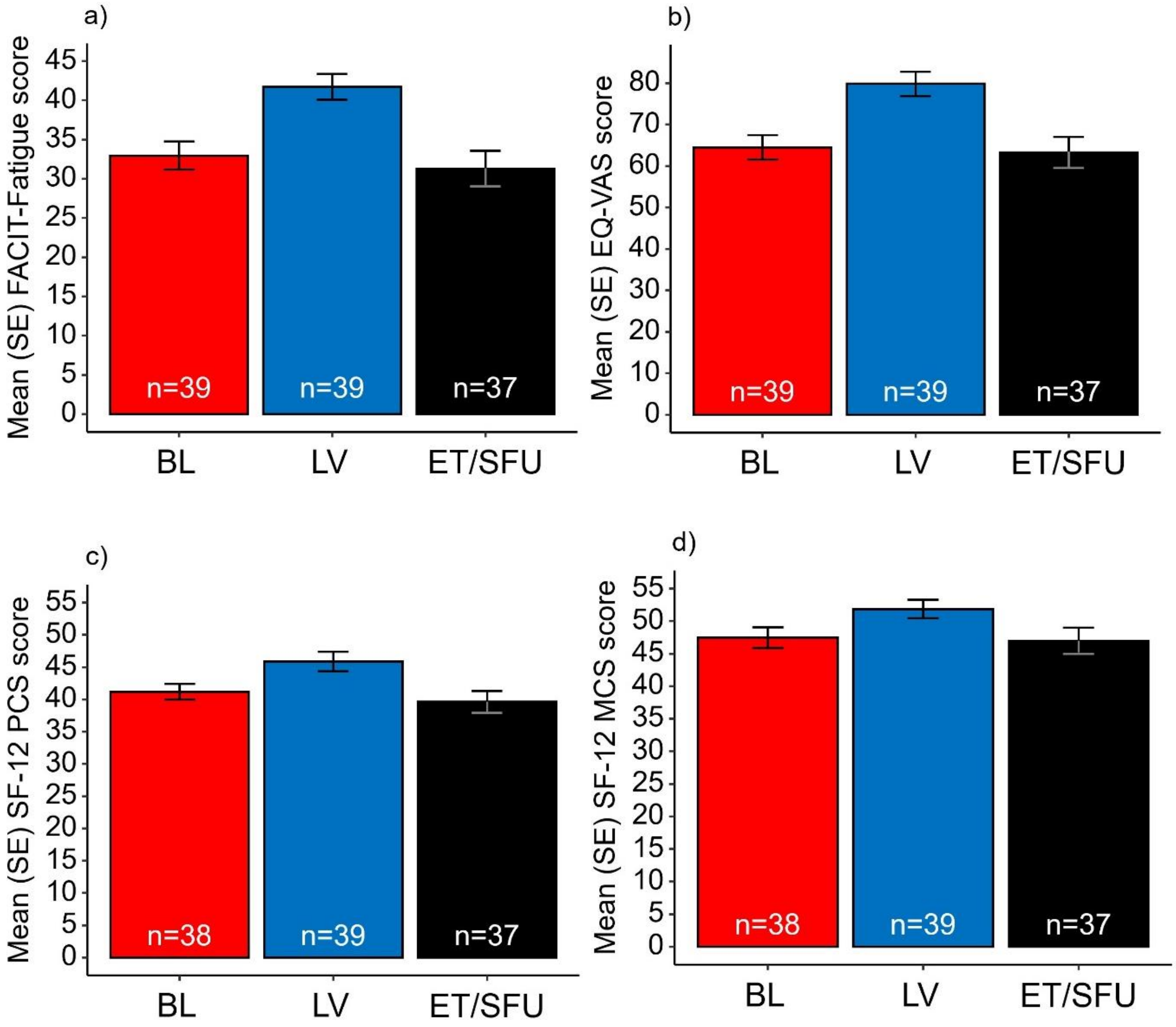


BL, baseline; EQ-VAS, EuroQol visual analogue scale; ET/SFU, early termination/safety follow-up 9 weeks post last sutimlimab dose; FACIT, Functional Assessment of Chronic Illness Therapy; LV, last on-treatment visit with available assessment; MCS, mental component score; PCS, physical component score; PRO, patient-reported outcome; SE, standard error; SF-12, short-form 12-item health survey.
